# Protocol: low cost fast and efficient generation of molecular tools for small RNA analysis

**DOI:** 10.1186/s13007-020-00581-w

**Published:** 2020-03-20

**Authors:** Diego López-Márquez, Ángel Del-Espino, Eduardo R. Bejarano, Carmen R. Beuzón, Javier Ruiz-Albert

**Affiliations:** Dpto. Biología Celular, Genética y Fisiología, Instituto de Hortofruticultura Subtropical y Mediterránea, Universidad de Málaga-Consejo Superior de Investigaciones Científicas (IHSM-UMA-CSIC), Campus de Teatinos, Málaga, 29071 Spain

**Keywords:** Vector, Target mimic, miRNA, Cloning, Plant, Downregulation

## Abstract

**Background:**

Small RNAs are sequence-dependent negative regulators of gene expression involved in many relevant plant processes such as development, genome stability, or stress response. Functional characterization of sRNAs in plants typically relies on the modification of the steady state levels of these molecules. State-of-the-art strategies to reduce plant sRNA levels include molecular tools such as Target Mimics (MIMs or TMs), Short Tandem Target Mimic (STTMs), or molecular SPONGES (SPs). Construction of these tools routinely involve many different molecular biology techniques, steps, and reagents rendering such processes expensive, time consuming, and difficult to implement, particularly high-throughput approaches.

**Results:**

We have developed a vector and a cloning strategy that significantly reduces the number of steps required for the generation of MIMs against any given small RNA (sRNA). Our pGREEN-based binary expression vector (pGREEN-DLM100) contains the *IPS1* gene from *A. thaliana* bisected by a *ccdB* cassette that is itself flanked by restriction sites for a type IIS endonuclease. Using a single digestion plus a sticky-end ligation step, the *ccd*B cassette that functions as a negative (counter) selection system is replaced by a pair of 28 nt self-annealing primers that provide specificity against the selected target miRNA/siRNA. The method considerably reduces the number of steps and the time required to generate the construct, minimizes the errors derived from long-range PCRs, bypasses bottlenecks derived from subcloning steps, and eliminates the need for any additional cloning technics and reagents, overall saving time and reagents.

**Conclusions:**

Our streamlined system guarantees a low cost, fast and efficient cloning process that it can be easily implemented into high-throughput strategies, since the same digested plasmid can be used for any given sRNA. We believe this method represents a significant technical improvement on state-of-the-art methods to facilitate the characterization of functional aspects of sRNA biology.

## Background

Gene silencing is a regulatory mechanism induced by the presence of double-stranded RNA (dsRNA) that mediates sequence specific downregulation of gene expression. In plants, gene silencing occurs through either inhibition of transcription by regulation of chromatin compaction (transcriptional gene silencing, TGS), or by degradation or inhibition of mRNA translation (post-transcriptional gene silencing, PTGS). These two mechanisms require the formation of small RNAs (sRNAs) of 21 to 24 nucleotides generated from dsRNA molecules, by Dicer or Dicer-like (DCL) proteins [1]. In plants, endogenous sRNAs are placed into two distinct groups, microRNAs (miRNAs) and small interfering RNA (siRNAs), which function at both transcriptional and post-transcriptional levels. MiRNAs originate from primary transcripts (pri-miRNAs), which fold into hairpin-like structures, while siRNAs derive from dsRNA precursors. SiRNAs can be divided into three subclasses: (i) heterochromatic siRNAs (hetsiRNAs), produced from repetitive regions or transposons, which regulate gene expression at transcriptional level; and (ii) secondary siRNAs, generated from dsRNA produced by replication of single stranded RNA (ssRNA) by RNA-dependent RNA polymerases (RDRs), and (iii) natural antisense transcript siRNAs (natsiRNAs), generated from dsRNAs formed by hybridization of complementary and independently transcribed RNAs, both of which are mainly involved in post-transcriptional regulation [2].

Typical strategies to study the function of small RNAs in plants (miRNA and siRNA) include the modification of the steady state levels of these molecules. sRNA levels can be increased either by directly overexpressing the endogenous precursor [3] or by the use of molecular tools such as artificial miRNAs (amiRs), which substitutes the 21 nt sequence of an endogenous pri-miRNA for our miRNA of interest [4, 5]. Conversely, molecular tools such as Target Mimics (MIMs or TMs) [6, 7], Short Tandem Target Mimic (STTMs) [8, 9], or molecular SPONGES (SPs) [10] are used to reduce plant miRNA levels with varying degrees of efficacy, which depend mainly on the miRNA family targeted [10]. Such techniques are extensively employed in both model (*Arabidopsis*) and crop plants (e.g. tomato) [9–11].

MIMs are transcripts usually based on the *INDUCED BY PHOSPHATE STARVATION1* (*IPS1*) gene, which is purposely modified to contain a single non-cleavable binding site for the miRNA of interest [6, 7]. A typical MIM is about 500 nucleotides long, and its miRNA-binding site has been engineered with three central mismatches to render it non-cleavable. The miRNA of interest, against which the MIM has been designed, will specifically bind to this engineered binding site but, being unable to cleave it, will remain attached to the MIM and therefore will not be able to exert its regulatory function elsewhere, sometimes even triggering the degradation of the miRNA thus sequestered [11]. Such specific seizing of miRNA copies usually achieve downregulation (knock down) of miRNA function. STTMs are an evolution of the MIM concept, since they are comprised of two identical copies of the non-cleavable binding site for the miRNA of interest, linked by a weak stem-loop spacer that confers stability to the final structure [8]. A typical STTM is about 100 nucleotides long, with the stem loop of 48–88 nucleotides making for most of its length. For its application in plant systems, SP are synthetic transcripts that contain up to 15 copies of the binding site to the miRNA of interest, each one of them rendered non-cleavable by means of two central mismatches, thus their specific designation of cmSPs (central mismatch SP) [10]. In plant systems, MIMs and STTMs are usually selected as a more reliable choice when looking for a strong loss-of-function phenotype, while cmSPs might be considered as a complementary technology to use when the previous technologies fail to downregulate a given miRNA family [10].

Plant genomes present over 200 miRNA families (miRBase version 21; [12]), each one often comprising a number of miRNA copies with very similar yet not identical sequences (i.e. miR156 family has 10 copies in *Arabidopsis*, miRBase version 21), which are expressed from different loci with differing temporal or spatial patterns, and may display partially or fully redundant functions [13, 14]. Technologies such as MIMs, STTMs and SPs allow the researcher to achieve specific downregulation of all members of any given miRNA family. Although these technologies represent a significant step forward for the characterization of sRNA function, state-of-the-art cloning strategies to generate MIM or STTM expression vectors routinely involve many different molecular biology techniques, steps, and reagents rendering such processes expensive, time consuming, and difficult to implement [15, 16]. This is particularly relevant for high-throughput approaches, which require the generation of dozens or even hundreds of constructs [7, 9]. For instance, the generation of a single MIM construct requires three consecutive PCR reactions using two primers specific for the target miRNA, plus two generic primers for the *IPS1* gene, followed by two consecutive cloning steps [16]. The generation of STTM constructs is also complex, requiring two long-range PCR reactions using two primers specific for the miRNA targeted, plus two generic primers for the intermediate cloning vector designed for the system, and two independent cloning steps with conventional restriction enzymes [15].

In our lab, in an effort to streamline the generation of constructs for MIM or STTM and expedite the application of these molecular tools to high-throughput approaches, we have developed a vector and a cloning method that significantly reduces the number of steps required for the generation of MIMs against any given small RNA. For this purpose, we have developed pGREEN-DLM100, a pGREEN-based binary expression vector (35S; NOST; [17]) containing a modified version of the *IPS1* gene from *A. thaliana*, and harboring the *ccdB* cassette between two restriction sites of a type IIS endonuclease. In our streamlined system, using a single digestion plus a sticky-end ligation step, we replace the *ccd*B cassette that functions as a negative (counter) selection system by a couple of 28 nt self-annealing primers that provide specificity against the selected target sRNA. The method reduces considerably the number of steps and thus the time required to generate the construct, minimizes the errors derived from long-range PCRs, bypasses bottlenecks derived from subcloning steps, and eliminates the need for any of the above mentioned cloning technics and reagents, overall saving time and use of reagents. Direct selection of positive clones is achieved simply by transforming the ligation in CcdB-susceptible bacteria (e.g. DH5a) in medium supplemented with the corresponding antibiotic, since linearized plasmids not accepting the primer duplex will remain linearized due to the incompatible ends generated by the type IIS endonuclease, while those undigested and thus still carrying the *ccdB* cassette will be counter-selected. Here we present the vector and cloning method, and a proof-of-principle application using miR319 as target, a well described miRNA with easy-to-monitor plant phenotypes [7, 9, 18].

Our system guarantees a low cost, fast and efficient cloning process that can be easily implemented into high-throughput strategies, since the same digested plasmid can be used for any given miRNA/siRNA.

Since small RNAs are sequence-dependent negative regulators of gene expression involved in many relevant plant processes such as development, genome stability, or stress response (biotic and abiotic) [1, 19, 20], the proposed cloning strategy represents an straightforward tool to characterize many aspects related with small RNA biology, and as such could potentially become a valuable asset for the research community.

## Materials

### Reagents and solutions

- Lysogenic Broth (LB; [21])
- Tryptone (Oxoid Limited, UK, Cat. no. LP0042)
- Yeast extract (Panreac, Germany, Cat. no. 403687)
- Sodium chloride (Panreac Cat. no. 121659)
- Bacteriological agar (Panreac Cat. no. 402302)
- Sterile deionized water
- Kanamycin (Km; Sigma, USA, Cat. no. K4378)
- Rifampicin, (Rif; Duchefa Biochemie, Netherlands, Cat. no. R0146)
- Gentamycin (Gm; Sigma, Cat. no. G3632)
- Tetracycline (Tet; Sigma, Cat. no. T3383)
- EDTA (Sigma, Cat. no. E5134)
- TRIS (Panreac Cat. no. A1379)
- BsmBI enzyme and 10× Reaction Buffer (NEB, USA)
- T4 DNA ligase and 10× Reaction Buffer (TAKARA, Japan)
- CcdB-resistant bacteria (e.g. DB3.1; [22])
- CcdB-sensitive bacteria (e.g. DH5a; [23])
- *Agrobacterium tumefaciens* (e.g. GV3101; [24])

### Equipment

- Thermocycler
- 37 °C incubator
- 28 °C incubator
- Petri dishes

### Reagent setup

- Lysogenic Broth (LB; [21]): Dissolve 10 g of tryptone, 5 g of yeast extract and 5 g of NaCl into 800 ml of distilled water. Adjust the volume to 1 l with distilled water and autoclave at 121 °C for 20 min. For solid LB, before autoclaving add 16 g/l of bacteriological agar.
- Antibiotics: For *E. coli* strains carrying the pGREEN-DLM100 vector or derivatives: kanamycin (50 μg/ml). For *Agrobacterium* strains use: rifampicin (50 μg/ml), tetracycline (5 μg/ml), gentamycin (25 μg/ml), and kanamycin (50 μg/ml).
- 10× annealing buffer (1 ml): Mix 100 μl 1 M Tris pH 8.0, 500 μl of 1 M NaCl, 10 μl of 1 M EDTA and 390 μl of double distilled H_2_O.

### Protocol

The novel cloning protocol is fully finished by day 3, with the DNA extraction of the final MIM-carrying plasmids ready for transformation into *Agrobacterium*. Days 4 and 5 detail the transformation processes common to any MIM protocol, and are included here for reference.

### Primer design

Critical step: Errors in primer design would compromise the success of the entire technique. Two aspects are essential during design:

1. To succeed at the ligation stage both primers must contain 5′ overhangs that anneal with the sticky ends produced by BsmBI digestion. In our case, the Forward primer contains the sequence 5′-TTGG-3′ while the Reverse primer carries the 5′-AGCT-3′ sequence (Fig. 1a).

**Fig. 1:**
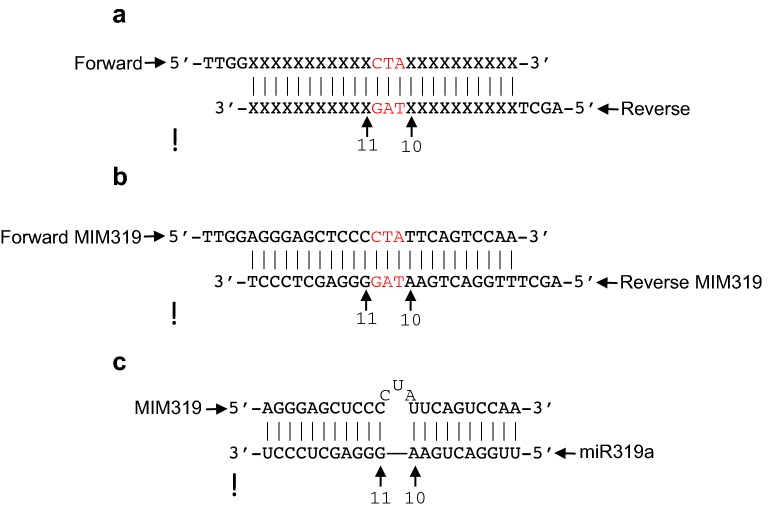
**a** A typical pair of self-annealing primers (forward and reverse) is shown. In red the “CTA” insertion. **b** As a representative case, the primer duplex used to generate MIM319 is shown. **c** Complementarity between MIM319 and miR319a from *Arabidopsis thaliana*. Nucleotides 10th and 11th of the miRNA are indicated with arrowheads
2. To correctly knockdown the desired sRNA, the Forward primer should include the reverse complementary sequence to the target sRNA, and harbor between positions 10th and 11th (relative to the sRNA sequence) a 3-nt insertion that will cause a bulge and consequently block the RISC-sRNA action. By default the “CTA” sequence is used (Fig. 1a) [7], unless the target sRNA of interest were to contain a “T” at position 11th, which would anneal with the “A” present in the “CTA” insertion, thus disturbing the function of the mimicry. If this was the case, the “CTA” sequence should be changed by a non-complementary sequence [7]. The Reverse primer should include the reverse complementary sequence to the Forward primer, plus the 5′ overhang defined above (Fig. 1a).

An example of primer design is shown in Fig. 1b, based on MIM319 and miR319a from *Arabidopsis thaliana*.

### Ligation and transformation

Day 1. Timing: 3–4 h

1. Carry out a plasmid prep of the *IPS*-*ccd*B containing plasmid (pGREEN-DLM100) from a CcdB-resistant bacterial strain (DB3.1). Use your preferred miniprep protocol. We used a rather standard classic miniprep protocol [25].
2. Digest the plasmid with the BsmBI enzyme. A typical reaction mix contains: 2.5 μg of *IPS*-*ccd*B containing plasmid, 5 μl of 10× reaction buffer, 2 units of BsmBI, and double distilled H_2_O to 50 μl. Incubate the reaction for 1 h at 55 °C. Inactivate the enzyme by incubating at 80 °C for 20 min (optional). The plasmid can be purified using a column to remove salts present in the restriction buffer (optional).
3. Save the digested plasmid. The digestion mix suggested above should render enough plasmid to generate 50 different MIM recombinant plasmids, facilitating the implementation of the protocol into high-throughput strategies.
4. While the digestion reaction takes place you may prepare your primers as follows: Mix 1 μl of Forward primer (100 μM), 1 μl of Reverse primer (100 μM), 1 μl of 10× annealing buffer (100 mM Tris pH 7.5–8.0, 500 mM NaCl, 10 mM EDTA) and 7 μl double distilled H_2_O. Use a thermocycler to heat the mix 5 min at 95 °C and then progressively cool it down to 25 °C at 0.1 °C/s. Dilute the annealed duplex 1:100 into water (this would render a 0.1 μM solution of the duplex). Alternatively, the mix can be heated to 95 °C 5 min and cooled down by incubating 1 h at room temperature. This step can be carried out with several primer couples in parallel, facilitating the implementation of the protocol into high-throughput strategies.
5. Set up the ligation step. For this purpose, mix 50 ng of BsmBI digested plasmid (1 μl), 1 μl of 0.1 μM diluted duplex, 1 μl T4 of DNA ligase, 1 μl 10× ligation buffer and 6 μl of double distilled H_2_O. Incubate 1 to 2 h at 16 °C. When in the generation of a single construct time is of the essence, the digestion and ligation steps (steps 3 and 5) can be carried out at once simultaneously in a single tube, in a manner similar to that described for Golden Gate cloning [26]. In such an event, preparation of the primers (step 4) should be carried out prior to the digestion/ligation step. For high-throughput strategies, the duplexes obtained (step 4) by the separate annealing of several primer couples are mixed in equal proportions, and 1 μl of this 0.1 μM mix is added to the ligation reaction.
6. Transform chemically competent CcdB-sensitive *E*. *coli* cells (e.g. DH5α) with 5 μl of the ligation product. Plate the transformation mix (100 μl and the volume obtained after concentrating it by centrifugation into a suitable volume for easy plating) onto Petri dishes containing solid LB medium supplemented with Km (50 μg/ml). Incubate overnight (ON) at 37 °C.

### Liquid culture for recombinant plasmid recovery

Day 2. Timing 10 min

1. Undigested plasmid should produce no colonies after transformation since the presence of CcdB toxin kills the bacteria, acting as a counter selection system [27, 28]. Select a couple of colonies and use them to inoculate 5 ml of LB supplemented with Km (50 μg/ml). Incubate ON at 37 °C with aeration.

### Plasmid extraction and *Agrobacterium* transformation

Day 3. Timing 2–3 h

1. Extract the plasmid from your ON cultures using your preferred miniprep protocol (see Day 1, step 1).
2. Check recombinant plasmids by sequencing. We typically send only a couple of candidates per construct since in our experience all recombinant plasmids sequenced displayed the correct expected sequence.
3. Transform GV3101 *Agrobacterium tumefaciens* competent cells [29] with 10–100 ng of the sequencing-checked recombinant plasmid. The *Agrobacterium* strain used should carry the pSOUP helper plasmid providing replication functions in trans for pGREEN [17]. Plate the transformation mixes onto Petri dishes containing solid LB medium supplemented with 50 μg/ml Rif, 5 μg/ml Tet, 25 μg/ml Gm, and 50 μg/ml Km. Incubate for 48 h at 28 °C to allow the growth of *Agrobacterium* colonies.

This is the last step of the cloning procedure. A side-by-side comparison of this improved procedure versus the standard is shown in Additional file 1: Fig. S2. The following steps are shared by any protocol requiring plant transformation.

### *Agrobacterium* starter culture

Day 5. Timing 10 min

1. Use the colonies obtained from the *A. tumefaciens* transformation plates to inoculate 5 ml of LB containing the appropriate antibiotic concentration (Rif, Tet, Gm, and Km), and incubate overnight at 28 °C with aeration.

### *Agrobacterium* liquid culture

Day 6. Timing 10 min

1. Use the starter culture to inoculate 100 ml of LB containing the appropriate antibiotic concentration (Rif, Tet, Gm, and Km), and incubate overnight at 28 °C with aeration.

### Plant transformation or *Nicotiana benthamiana* transient expression

Day 7. Timing 1 h

1. Centrifuge the ON culture at 4000*g* 10 min. Discard the supernatant, and suspend the precipitate into transformation media (5% sucrose and 0.05 Silwet L-77). Transform *A. thaliana* plants using the floral dipping method [30]. Alternatively, suspend the precipitate into infiltrating media (10 mM MES pH 5.6, 10 mM MgCl_2_ and 0.2 mM Acetosyringone) to generate the inoculum to infiltrated *N. benthamiana* leaves for transient expression [31].

**Fig. 2:**
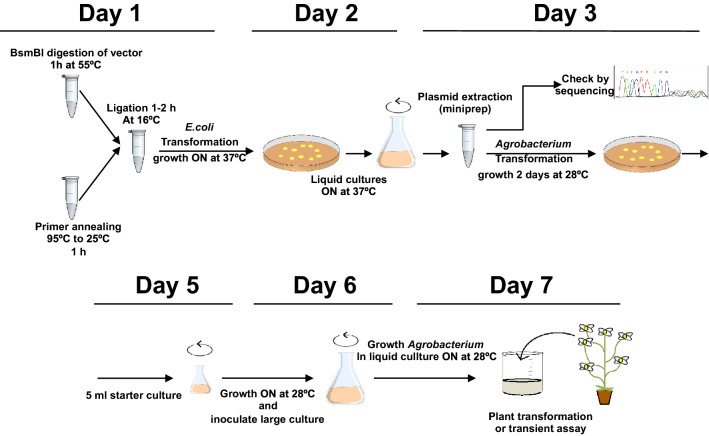
Schematic representation of the overall process

A schematic representation of the entire process is shown in Fig. 2.

## Results and discussion

### Highly efficient generation of targets mimics

Protocols for generation of MIMs, STTMs and CmSPs are labor and time-consuming and involve the use of expensive reagents like high-fidelity polymerases, pGEM-T/pDONOR vectors, TOPO cloning, LR reactions (see Additional file 1: Fig. S2) or even the services of gene synthesis companies (CmSPs), increasing the cost of the entire process and hindering their use for high-throughput approaches. The aim of this work was to reduce to the minimum the costs and time required for the generation of target mimics and to pave the way to its application to high-throughput strategies.

**Fig. 3:**
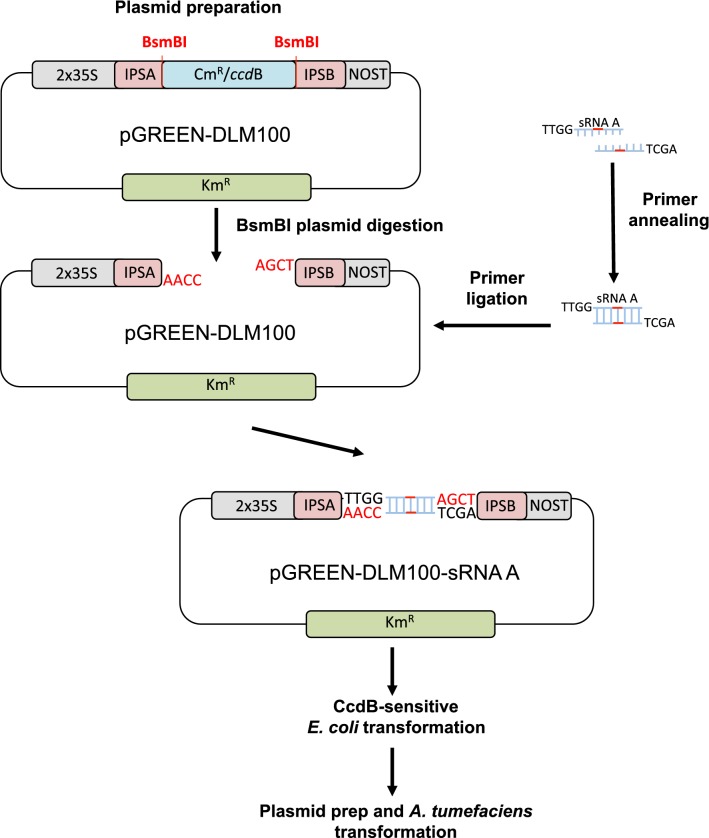
Schematic representation of primer cloning into the pGREEN-DLM100 vectors (protocol days 1–3)

**Fig. 4:**
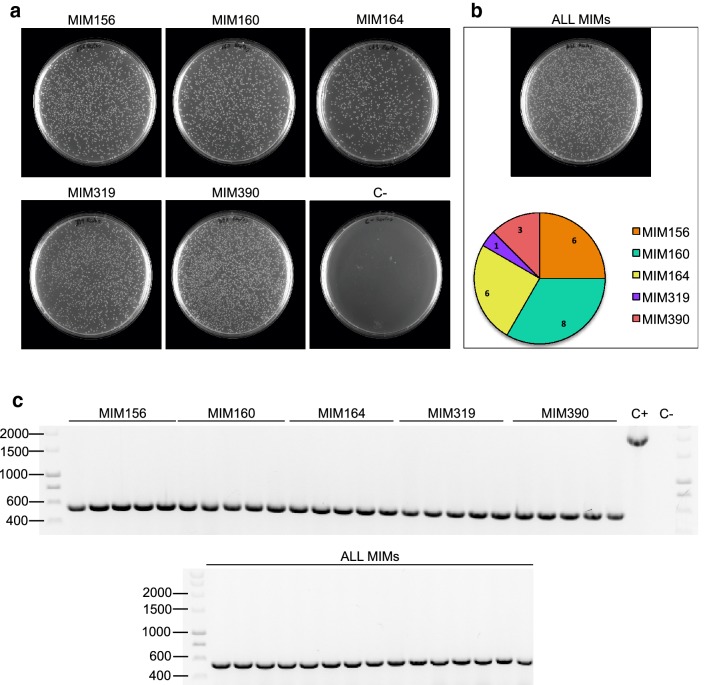
**a** Representative images showing the result of plating the full volume of each transformation into DH5α as obtained during the cloning process of individual MIMs (MIM156, MIM160, MIM164, MIM319 and MIM390). **b** In the top panel, a representative image showing the result of plating the full volume of the transformation into DH5α as obtained during the simultaneous cloning of all five MIMs within a single cloning reaction (mix of duplex pairs for MIM156, MIM160, MIM164, MIM319 and MIM390). In the bottom panel, the pie chart shows the distribution of clones obtained for each MIM construct among 24 colonies randomly selected from the plate shown in the top panel, as determined by sequencing. **c** The upper panel shows the PCR analysis of five colonies obtained from each of the plates from the individual MIM cloning process (**a**). The lower panel shows the PCR analysis of 15 colonies obtained from the plate corresponding to the simultaneous cloning of all five MIMs (**b**). All colonies were confirmed to have lost the *ccdB* cassette. C+ corresponds to the positive control reaction in which the original plasmid containing *ccdB* was used as a template in the PCR

For this goal, we have developed a pGREEN-based binary vector, pGREEN-DLM100 (for detailed information on its design and generation see Additional file 1: Fig. S1 and Methods), for the generation of target MIMICs constructs directly into a plant expression vector. In our system, the expression of the *IPS1*-based target MIMICs is under the control of two tandem copies of the cauliflower mosaic virus promoter (2× CaMV 35S) and this transcriptional unit is ended by a Nopaline Synthase terminator (tNOS) (Fig. 3 and Additional file 1: Fig. S1). The *IPS1* backbone was modified to harbor a Cm^R^/*ccdB* cassette flanked by two restriction sites for the type IIS restriction enzyme BsmBI (Fig. 3 and Additional file 1: Fig. S1). Type IIS restriction enzymes recognize asymmetric DNA sequences and cleave outside of their recognition sequence, generating 5′ or 3′ DNA overhanging ends that can include any nucleotide. Such enzymes have been used previously in various cloning strategies, such as Golden Gate cloning [26]. In our plasmid, digestion with BsmBI results in a linearized vector with two four-nucleotide 5′ overhanging ends that are not complementary to each other, and therefore incompatible. Moreover, undigested plasmids that could have remained throughout the process up to the transformation step would be counter-selected due to the presence of the *ccdB* gene. Such negative selection improves the efficiency of the process by reducing the background of colonies carrying non-recombinant plasmids, without the need of purifying the digested plasmid before the ligation step. Any given couple of previously self-annealed primers (conferring specificity against a target sRNA) can be directionally cloned into the linearized vector, as long as the sequences 5′-TTGG-3′ (Forward primer) and 5′-AGCT-3′ (Reverse primer) have been included into the corresponding 5′ primer ends (Figs. 1 and 3). In our system, a typical 1 h-long restriction followed by a 2-h ligation reaction allows the generation of the binary vector carrying a MIM construct against the miRNA of interest within just 1 day, and it does so with a very high efficiency (Fig. 4), substantially reducing the time and reagents required in other methods. While differences in cloning efficiency for specific sequences can not be ruled out, the cloning procedure does not seem to be particularly affected by the target sequence, since we generated five different MIM constructs (MIM156, MIM160, MIM164, MIM319, MIM390) for the corresponding *Arabidopsis* miRNAs, obtaining similar efficiencies (over one thousand colonies) for each independent cloning event (Fig. 4). Furthermore, the procedure is suitable for high-throughput approaches: as a proof of principle, we generated the same five MIM constructs simultaneously in a single reaction (Fig. 4); the upper limit of different duplexes that can be cloned simultaneously remains to be established. The CcdB-expressing cassette, acting as a counter selection system [27, 28], allows the efficient selection of recombinant plasmids when the ligation is directly transformed into a CcdB-sensitive bacteria (DH5α), since all bacteria carrying non-recombinant plasmids will not grow. To confirm this, we analyzed by PCR and/or sequencing (Fig. 4) five random clones out of each of the five independent cloning reactions, plus 24 random clones from the simultaneous cloning reaction: all sequenced clones carried the miRNA non-cleavable binding site engineered into the sequence of the corresponding primer duplex. In addition, BsmBI restriction of just 2.5 μg of our plasmid renders sufficient linearized vector for the individual generation of at least 50 different target MIMICs (50 ng per construct), or for 250 if MIMICs are generated in pools of five as shown here, which allows for the implementation of the protocol into high-throughput strategies.

### Functional validation of pGREEN-DLM100 derived target MIMICs

**Fig. 5:**
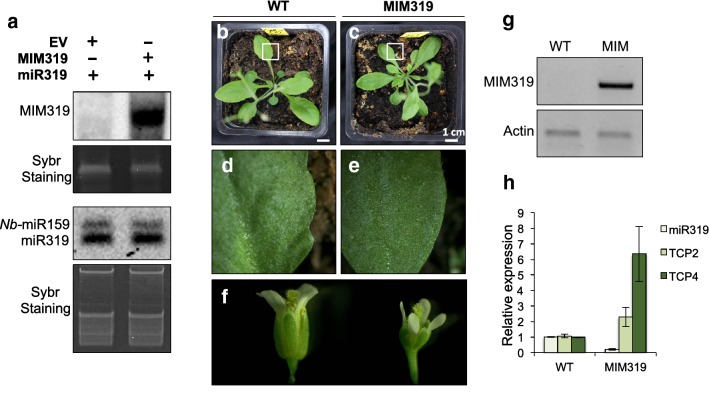
**a** Northern blot analysis of MIM319 (*IPS1* probe, upper panels) and miR319 (miR319 probe; lower panels) of *N. benthamiana* leaves transiently expressing the empty vector, the MIM319 construct, and/or the miR319 as indicated. Endogenous *N. benthamiana* miR159 can also be detected using the miR319 probe. Sybr (Thermo Scientific, USA) staining images, used to confirm equal loading, are shown below the corresponding Northern blots. Photographs showing whole plants and leaf detail of wild type Col-0 *Arabidopsis* plants (**b** and **d**) and transgenic plants expressing the MIM319 construct (**c** and **e**). **f** Flowers from wild type Col-0 *Arabidopsis* plants (left) and transgenic plants expressing the MIM319 construct (right). **g** Semi-quantitative RT-PCR showing accumulation of the MIM319 non-coding RNA (ncRNA) in inflorescences from MIM319 transgenic lines. Actin was used as an internal control. **h** RT-*q*PCR showing the relative levels of miR319 and two target genes (*TCP2* and *TCP4*) in inflorescences from wild type (WT) and MIM319 plants. The average and standard error of three technical replicates is represented

As a proof of principle of the efficiency of our pGREEN-based vector, we have chosen the target MIMIC against *A. thaliana* miR319a for further validation (Figs. 1, 4). The construct obtained (MIM319), an artificial non-coding RNA with a noncleavable miRNA319 recognition site, was sequenced-checked and transformed into *A. tumefaciens* (GV3101) and used for *Agrobacterium*-mediated transient expression in *N. benthamiana* leaves. High levels of MIM319 were detected by Northern blot analysis 2 days after inoculation, confirming the correct expression of the *IPS1*-modified transcript (Fig. 5a, upper panel). In parallel, miR319 (35S::miR319) was co-expressed either with the MIM319 construct or with the empty vector as a control. Similar levels of miR319 were detected in both scenarios, indicating that MIM319 expression does not result in the degradation of its target miR319 in our *Agrobacterium*-mediated transient assay, perhaps due to the disproportionate levels of miRNA produced in such assays. Downregulation of miRNA function in the absence of detectable miRNA degradation has been proposed to be due to sequestration of the miRNA via its interaction with the MIM/STTM construct [6, 9].

**Fig. 6:**
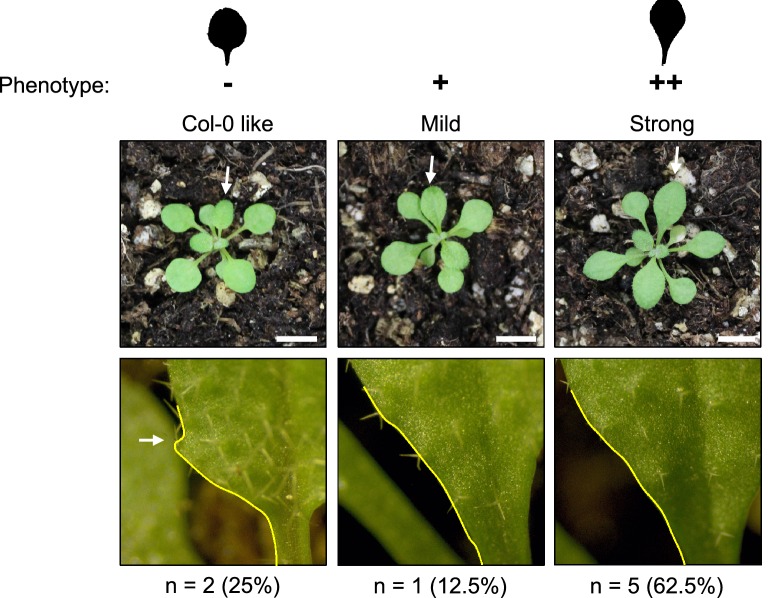
Phenotypic range of the MIM319 transgenic lines obtained in a typical transformation. Upper panels show images of whole 3 week-old transgenic plants displaying either wild type-like, mild or strong phenotype. A schematic view of typical leaves within the extreme phenotypic groups is shown on top. A close up view of leaves indicated with a white arrow in the upper panels is shown in the lower panels. The outline of the leave edge and the angle at the boundary between the leave and the petiole is indicated in yellow. The number of plants displaying each phenotype and the percentage they represent of the total number of transgenic plants characterized is shown below the lower panel for each phenotype

To confirm that the MIM construct generated using our system does indeed downregulate miRNA319 function, we used the MIM319 plasmid to generate *A. thaliana* transgenic lines through the floral dipping method [30]. Transgenic lines harboring the 2× 35S-MIM319 showed defective leaf development, displaying a lanceolated leaf shape and reduced leaf margin serration compared with WT plants (Fig. 5b, d versus c, e), as well as smaller flowers (Fig. 5f). All these phenotypes have been previously reported to be associated with either reduced levels of miR319 (MIM319; STTM319; mir319a/b) or expression of *rTCP4*, a miR319-resistant version of one of the main targets of this miRNA [7, 9, 18, 32]. In addition, we confirmed the accumulation of the MIM319 non-coding RNA (ncRNA) in MIM319 transgenic lines, by semi-quantitative RT-PCR (Fig. 5g), and also determined the levels of miR319 and two of its target genes, *TCP2* and *TCP4* [33] using RT-qPCRs on samples taken from wild-type and MIM319 inflorescences. This analysis showed a reduction of close to 90% in miR319 levels in MIM319 plants, and a concomitant increase in the levels of both target genes (Fig. 5h), in agreement with previous reports [34]. The range of phenotypic variation displayed by the transformants is shown in Fig. 6. These results validate the efficiency and specificity of MIM constructs to downregulate target miRNA/siRNA function generated with our optimized fast and easy cloning system.

## Supplementary information

**Additional file 1.** Supplementary methods, figures and table.

## Data Availability

All data generated or analyzed during this study are included in this published article and its additional information files.

## References

[1] Borges F, Martienssen RA (2015). The expanding world of small RNAs in plants. Nat Rev Mol Cell Biol.

[2] Axtell MJ (2013). Classification and comparison of small RNAs from plants. Annu Rev Plant Biol.

[3] Llave C, Xie Z, Kasschau KD, Carrington JC (2002). Cleavage of scarecrow-like mRNA targets directed by a class of Arabidopsis miRNA. Science.

[4] Schwab R (2006). Highly specific gene silencing by artificial MicroRNAs in Arabidopsis. Plant Cell.

[5] Ossowski S, Schwab R, Weigel D (2008). Gene silencing in plants using artificial microRNAs and other small RNAs. Plant J.

[6] Franco-Zorrilla JM, Valli A, Todesco M, Mateos I, Puga MI, Rubio-Somoza I (2007). Target mimicry provides a new mechanism for regulation of microRNA activity. Nat Genet.

[7] Todesco M, Rubio-Somoza I, Paz-Ares J, Weigel D (2010). A collection of target mimics for comprehensive analysis of MicroRNA function in *Arabidopsis thaliana*. PLoS Genet.

[8] Yan J, Gu Y, Jia X, Kang W, Pan S, Tang X (2012). Effective small RNA destruction by the expression of a short tandem target mimic in Arabidopsis. Plant Cell.

[9] Peng T, Qiao M, Liu H, Teotia S, Zhang Z, Zhao Y (2018). A resource for inactivation of MicroRNAs using short tandem target mimic technology in model and crop plants. Mol Plant.

[10] Reichel M, Li Y, Li J, Millar AA (2015). Inhibiting plant microRNA activity: molecular SPONGEs, target MIMICsand STTMs all display variable efficacies against target microRNAs. Plant Biotechnol J.

[11] Todesco M, Rubio-Somoza I, Paz-Ares J, Weigel D (2010). A collection of target mimics for comprehensive analysis of MicroRNA function in *Arabidopsis thaliana*. PLoS Genet.

[12] Axtell MJ, Meyers BC (2018). Revisiting criteria for plant MicroRNA annotation in the era of big data. Plant Cell.

[13] Tang G (2010). Plant microRNAs: an insight into their gene structures and evolution. Semin Cell Dev Biol.

[14] Cui J, You C, Chen X (2017). The evolution of microRNAs in plants. Curr Opin Plant Biol.

[15] Tang G, Yan J, Gu Y, Qiao M, Fan R, Mao Y (2012). Construction of short tandem target mimic (STTM) to block the functions of plant and animal microRNAs. Methods.

[16] Villar-Martin Luis Manuel, Rubio-Somoza Ignacio (2019). Mimicry Technology: A Versatile Tool for Small RNA Suppression. Methods in Molecular Biology.

[17] Hellens RP, Edwards EA, Leyland NR, Bean S, Mullineaux PM (2000). pGreen: a versatile and flexible binary Ti vector for. Plant Mol Biol.

[18] Koyama T, Sato F, Ohme-Takagi M (2017). Roles of miR319 and TCP transcription factors in leaf development. Plant Physiol.

[19] Brant EJ, Budak H (2018). Plant small non-coding RNAs and their roles in biotic stresses. Front Plant Sci.

[20] Sunkar R, Chinnusamy V, Zhu J, Zhu J-K (2007). Small RNAs as big players in plant abiotic stress responses and nutrient deprivation. Trends Plant Sci.

[21] Bertani G (1951). Studies on lysogenesis. I. The mode of phage liberation by lysogenic *Escherichia coli*. J Bacteriol.

[22] Bernard P, Couturier M (1992). Cell killing by the F plasmid CcdB protein involves poisoning of DNA-topoisomerase II complexes. J Mol Biol.

[23] Hanahan D (1983). Studies on transformation of *Escherichia coli* with plasmids. J Mol Biol.

[24] Holsters M, Silva B, Van Vliet F, Genetello C, De Block M, Dhaese P (1980). The functional organization of the nopaline *A. tumefaciens* plasmid pTiC58. Plasmid.

[25] Stephen D, Jones C, Schofield JP (1990). A rapid method for isolating high quality plasmid DNA suitable for DNA sequencing. Nucleic Acids Res.

[26] Engler C, Kandzia R, Marillonnet S (2008). A one pot, one step, precision cloning method with high throughput capability. PLoS ONE.

[27] Bernard P, Gabant P, Bahassi EM, Couturier M (1994). Positive-selection vectors using the F plasmid ccdB killer gene. Gene.

[28] Bernard P (1995). New ccdB positive-selection cloning vectors with kanamycin or chloramphenicol selectable markers. Gene.

[29] McCormac AC, Elliott MC, Chen DF (1998). A simple method for the production of highly competent cells of Agrobacterium for transformation via electroporation. Mol Biotechnol.

[30] Clough SJ, Bent AF (1998). Floral dip: a simplified method for Agrobacterium-mediated transformation of *Arabidopsis thaliana*. Plant J.

[31] Zheng X, Li X, Wang B, Cheng D, Li Y, Li W (2019). A systematic screen of conserved *Ralstonia solanacearum* effectors reveals the role of RipAB, a nuclear-localized effector that suppresses immune responses in potato. Mol Plant Pathol.

[32] Schommer C, Debernardi JM, Bresso EG, Rodriguez RE, Palatnik JF (2014). Repression of cell proliferation by miR319-regulated TCP4. Mol Plant.

[33] Palatnik JF, Allen E, Wu X, Schommer C, Schwab R, Carrington JC (2003). Control of leaf morphogenesis by microRNAs. Nature.

[34] Rubio-Somoza I, Weigel D (2013). Coordination of flower maturation by a regulatory circuit of three MicroRNAs. PLoS Genet.

