# Supplementary material for: Protocol: low cost fast and efficient generation of molecular tools for small RNA analysis
Source: Plant Methods. 2020 Mar 20;16:41. doi: 10.1186/s13007-020-00581-w (PMC7082952; doi:10.1186/s13007-020-00581-w)
Supplement: Supplementary file 1 — Additional file 1. Supplementary methods, figures and table. [file 13007_2020_581_MOESM1_ESM.pdf]

pGREEN-DLM100 vector construction

First, a BsmBI site present in plasmid pGreen0029[1] was removed by long range inverted PCR using the NZYMutagenesis Kit (NZYTech, Portugal) and the BsmBI-Km-removeA/BsmBI-Km-removeB primers. The Cm^R-*ccdB* cassette was PCR-amplified from pGWB6[2], using the primers Ccdb1/Ccdb2 containing the BsmBI sites. On the other hand, the IPSA and IPSB fragments were amplified from the *Arabidopsis thaliana* genome using IPSA1/IPSA2 and IPSB1/IPSB2 primers, respectively, and fused to the Cm^R-*ccdB* cassette by overlapping PCR, giving rise to the IPSA- Cm^R/*ccdB*-IPSB fragment. Then, the 2x35S promoter and Nos terminator were PCR-amplified from a pBINX' plasmid[3], using 35S-F/35S-R and NosT-F/NosT-R primers, respectively, and fused to IPSA-Cm^R/*ccdB*-IPSB fragment via overlapping PCR to produce the final DNA fragment containing 2x35S-IPSA-Cm^R/*ccdB*-IPSB-NosT. Afterwards, this PCR product was ligated into the EcoRV site of the pGreen0029 derivative lacking the BsmBI site generated previously, and transformants were selected on LB plates supplemented with Cm. Finally, an inverted PCR was performed on the recombinant plasmid obtained to remove a BsmBI site present in the Cm^R-*ccdB* cassette, using primers BsmBI-Ccdb-removeA/BsmBI-Ccdb-removeB and the NZYMutagenesis Kit. All primers used in this study are detailed in Supplementary Table S1. Unless otherwise stated, PCRs were performed using Q5 High-Fidelity DNA Polymerase (NEB, USA) following the manufacturer's instructions. A graphic overview of the process is included in Fig. S1.

RNA extraction and Northern blot

Total RNA was extracted as previously described[4] or using TRISURE (Bioline, UK) for RT-*q*PCRs. Northern blot analyses were carried out as previously described in [5], with some modifications. In brief, for low molecular weight Northern blot analyses used to detect miR319, 10 μg of total RNA was suspended into 2x RNA loading buffer (95% formamide, 18 mM EDTA pH 8.0, 0.025% sodium dodecyl sulfate (SDS), 0.01% bromophenol blue, 0.01% xylene cyanol) and denatured at 90ºC for 5 min. Afterwards, samples were analysed in a 7M urea, 0.5X TBE, 17% polyacrylamide gel run at 180 V until bromophenol blue runs out of the gel. Then, RNA was electro-blotted onto an Amersham

Hybond-N^+ nylon membrane (GE Healthcare Life Sciences, USA) at 80 V for 1 hour in cold 0.5x TBE. After transfer, RNA was fixed onto the membrane using UV light (0.120 J) and dried for 1 h at 80ºC. After this step, the membrane was pre-hybridize in Church buffer (1% BSA, 1 mM EDTA, 0.5 M phosphate buffer, 7% SDS) for 1 h at 40ºC. Hybridization was carried out using the same buffer with the added probe and incubated overnight at 40ºC. The next day, the membrane was washed 4 times with a 2x SSC, 0.1% SDS solution at 40ºC (10 min per wash) and detection was carried out as previously described by [5]. For detection of the *IPS1* transcript, we used the same protocol described above with samples separated into a 7M urea, 0.5X TBE, 5% polyacrylamide gel.

cDNA synthesis, RT-*q*PCRs and semi-quantitative PCRs

For quantification of mature miRNAs, we used the stem-loop RT-*q*PCR method [6]. Pulsed-RT (1 step at 16ºC of 30 min, followed by 60 cycles at 30ºC for 30 s, 42ºC for 30 s and 50ºC for 1 min, and 1 cycle of 85ºC for 5 min) was performed using Revert Aid First Strand cDNA Synthesis Kit (Thermo Fisher Scientific, USA) with an specific RT stem-loop primer for miR319, and oligo (dT) primers. Stem-loop RT-*q*PCRs were performed in a CFX96 detection system (Bio-Rad, USA) following a previously described protocol [6], using 2 µl of a 1/2 dilution of the cDNA generated previously. Primers used to detect miR319 are listed in Supplementary Table S1.

For each of the *TCP2*, *TCP4* and actin RT-*q*PCRs, we added 2 µl of a 1 in 2 dilution of the cDNA generated previously, to a reaction containing 5 µl of SsoFast EvaGreen (Bio-Rad, USA), 0.5 µl of each of the corresponding Forward and Reverse primers (10 µM), and 2 µl of H2O. RT-*q*PCRs were performed using a CFX96 detection system (Bio-Rad, USA) with a first denaturing step at 95ºC 1 min, followed by 40 cycles of 95ºC 10 s and 60ºC 17 s. In all cases actin was used as internal control. Relative expression was calculated using the $2^{-\Delta\Delta Ct}$ method [7]. Primers used to detect *TCP2*, *TCP4* and actin are listed in Supplementary Table S1.

For semi-quantitative PCRs, we used 2 µl of the cDNA generated previously, and a non-saturating number of cycles (22 cycles). PCRs were performed using GoTaq DNA Polymerase (PROMEGA, USA) following the manufacturer's

instructions. Primers IPSA1 and MIM319-R (Supplementary Table S1) were used for MIM319 detection.

Probe labelling

For detection of miR319, a DNA reverse complement primer to miR319a sequence was 3'-end-labelled with Digoxigening-11-ddUTP (Sigma, USA) using a Terminal Deoxynucleotidyl Transferase (TdT; ThermoFisher SCIENTIFIC, USA) in a reaction containing: 20 Units TdT, 10 μl 5x TdT reaction buffer, 5 μl DNA primer (1 μM), 2.5 μl Digoxigenin-11-ddUTP (10 μM) and bidistilled H_2O to 50 μl. The reaction was incubated for 40 min at 37ºC and directly added to the hybridization solution without further purification.

For detection of *IPS1*, endogenous *Arabidopsis IPS1* transcript was PCR-amplified using Q5 High-Fidelity DNA Polymerase (NEB, USA) with IPSA1 and IPSB2 as primers. Then, PCR product was gel-purified and used as a template in a random priming reaction containing: 400 ng DNA template, 4 Units Klenow fragment (TAKARA, Japan), 5 μl 10x Klenow reaction buffer, 12 μl Random hexamers (100 μM), 5 μl 10x PCR DIG labelling Mix (Sigma, USA) and H_2O to 50 μl. The reaction was incubated 4 h at 37ºC and directly added to the hybridization solution without additional purification.

Supplementary figure legends

Figure S1: Schematic representation of the cloning process carried out to generate the pGREEN-DML100 vector.

Figure S2: Comparative between the classical cloning protocol for MIM generation and our streamlined method.

References

1. Hellens RP, Edwards EA, Leyland NR, Bean S, Mullineaux PM. pGreen: a versatile and flexible binary Ti vector for. Plant Mol Biol. Kluwer Academic Publishers; 2000;42(6):819–32.

2. Nakagawa T, Suzuki T, Murata S, Nakamura S, Hino T, Maeo K, et al. Improved Gateway Binary Vectors: High-Performance Vectors for Creation of Fusion Constructs in Transgenic Analysis of Plants. Bioscience, Biotechnology, and Biochemistry. 2014 May 22;71(8):2095–100.

3. Sánchez-Durán MA, Dallas MB, Ascencio-Ibañez JT, Reyes MI, Arroyo-Mateos M, Ruiz-Albert J, et al. Interaction between geminivirus replication protein and the SUMO-conjugating enzyme is required for viral infection. J Virol. 3rd ed. 2011 Oct;85(19):9789–800.

4. Couto D, Stransfeld L, Arruabarrena A, Zipfel C, Lozano-Durán R. Broad application of a simple and affordable protocol for isolating plant RNA. BMC Res Notes. BioMed Central; 2015 Apr 16;8(1):154–3.

5. Tomassi AH, Gagliardi D, Cambiagno DA, Manavella PA. Nonradioactive Detection of Small RNAs Using Digoxigenin-Labeled Probes. MicroRNAs in Development. New York, NY: Springer New York; 2017;1640(18):199–210.

6. Varkonyi-Gasic E. Stem-Loop qRT-PCR for the Detection of Plant microRNAs. In: MicroRNAs in Development. Boston, MA: Springer US; 2016. pp. 163–75. (Methods in Molecular Biology; vol. 1456).

7. Livak KJ, Schmittgen TD. Analysis of Relative Gene Expression Data Using Real-Time Quantitative PCR and the 2−ΔΔCT Method. Methods. 2001 Dec;25(4):402–8.

Primers used in this study

Name	Sequence (5'-3')	Used for
IPSA1	GCAGGTCGACCAAACACCACAAAAACAAAAG	Cloning of IPSA fragment
IPSA2	AATGCGGCCCGTCTCCCCAATTTCTAGAGGGAGATA	Cloning of IPSA fragment
Ccdb1	GAAATTGGGGAGACGGGCCGCATTAGGCACCCCAG	Cloning of *ccdB* cassette
Ccdb2	CCGAAGCTTGAGACGTGCAGACTGGCTGTGTATAAG	Cloning of *ccdB* cassette
IPSB1	CAGTCTGCACGTCTCAAGCTTCGGTTCCCCTCGGA	Cloning of IPSB fragment
IPSB2	GCTCGGTACCAAGAGGAATTCACTATAAAGAGAATC	Cloning of IPSB fragment
BsmBI-Ccdb-removeA	ATGTTTTTCGTATCAGCCAATCCCTGGGTGAGTTTCACCAGTTTTG	Removal a BsmBI site from *ccdB* cassette
BsmBI-Ccdb-removeB	GATTGGCTGATACGAAAAACATATTCTCAATAAACCCTTTAGGGAAATAG	Removal a BsmBI site from *ccdB* cassette
35S-F	CCATGATTACGCCAAGCTGG	Cloning of 2x35S promoter
35S-R	TTGTGGTGTTTGGTCGACCTGCAGGCATGCGTTAAC	Cloning of 2x35S promoter
NosT-F	AGTGAATTCCTCTTGGTACCGAGCTCAGATCTCAGC	Cloning of NOS terminator
NosT-R	ACGGCCAGTGAATTGTTAATTAAG	Cloning of NOS terminator
BsmBI-Km-removeA	GCGTATTTCGCCTCGCTCAGGCGCAATCACGAATGAATAACGG	Removal a BsmBI site from Km

BsmBI-Km-removeB	CCTGAGCGAGGCGAAATACGCGATCGCTGTTAAAAGGACAATTAC	Removal a BsmBI site from Km
MIM319-F	TTGGAGGGAGCTCCCCTATTCAGTCCAA	Generation of MIM319A plasmid
MIM319-R	AGCTTTGGACTGAATAGGGGAGCTCCCT	Generation of MIM319A plasmid
miR319a_probe	AGGGAGCTCCCTTCAGTCCAA	Probe for Northern blot
MIM156-F	TTGGGTGCTCACTCCTATCTTCTGTCA	Generation of MIM156 plasmid
MIM156-R	AGCTTGACAGAAGATAGGAGTGAGCAC	Generation of MIM156 plasmid
MIM160-F	TTGGTGGCATACAGGCTAGAGCCAGGCA	Generation of MIM160 plasmid
MIM160-R	AGCTTGCCTGGCTCTAGCCTGTATGCCA	Generation of MIM160 plasmid
MIM164-F	TTGGTGCACGTGCCCCTATGCTTCTCCA	Generation of MIM164 plasmid
MIM164-R	AGCTTGGAGAAGCATAGGGGCACGTGCA	Generation of MIM164 plasmid
MIM390-F	TTGGGGCGCTATCCCCTATCCTGAGCTT	Generation of MIM390 plasmid
MIM390-R	AGCTAAGCTCAGGATAGGGGATAGCGCC	Generation of MIM390 plasmid
Actin-F	GGCAAGTCATCACGATTGG	Normalization of RT- *q* PCR
Actin-R	CAGCTTCCATTCCCACAAAC	Normalization of RT- *q* PCR
RT stem loop miR319	GTCGTATCCAGTGCAGGGTCCGAGGTATTCGCACTGGATACGACAGGGAG	For pulsed RT and cuantification of miR319
Reverse Universal stem loop	GTGCAGGGTCCGAGGT	Cuantification of miRNAs by RT-*q* PCR
Forward miR319	TGGCGTTGGACTGAAGGGAG	Cuantifiation of miR319 by RT-*q* PCR
TCP2-F	AACGGCGGAGCATTCAATCTT	Cuantifiation of TCP2 by RT-*q* PCR
TCP2-R	GCCTTTACCCTTATGTTCTGA	Cuantifiation of TCP2 by RT-*q* PCR
TCP4-F	CCTTCAACGACGTCGTTTCAGCCAG	Cuantifiation of TCP4 by RT-*q* PCR
TCP4-R	GTGAACCGGTGGAGGAAGGTGATG	Cuantifiation of TCP4 by RT-*q* PCR

Vector Generation

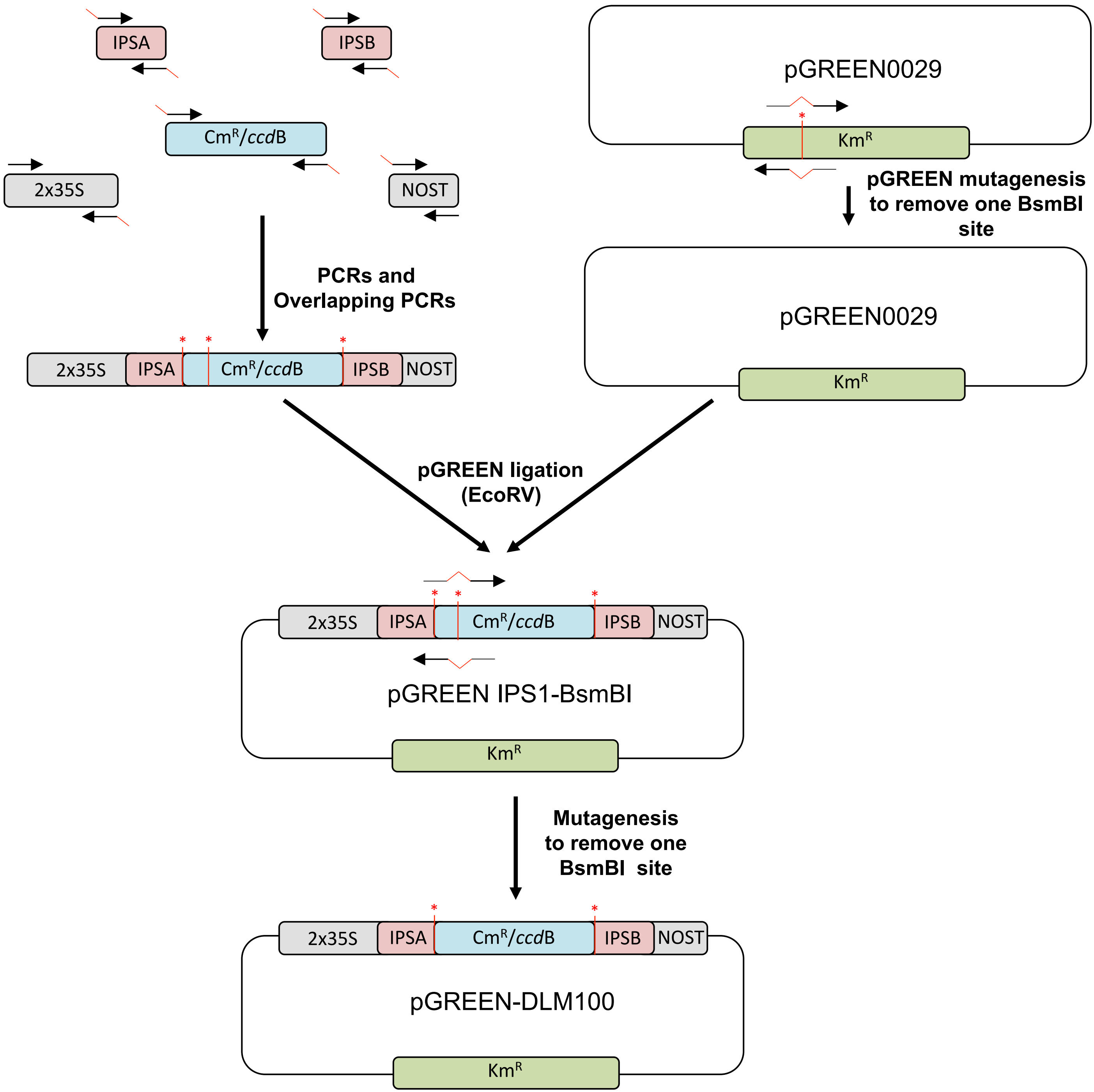

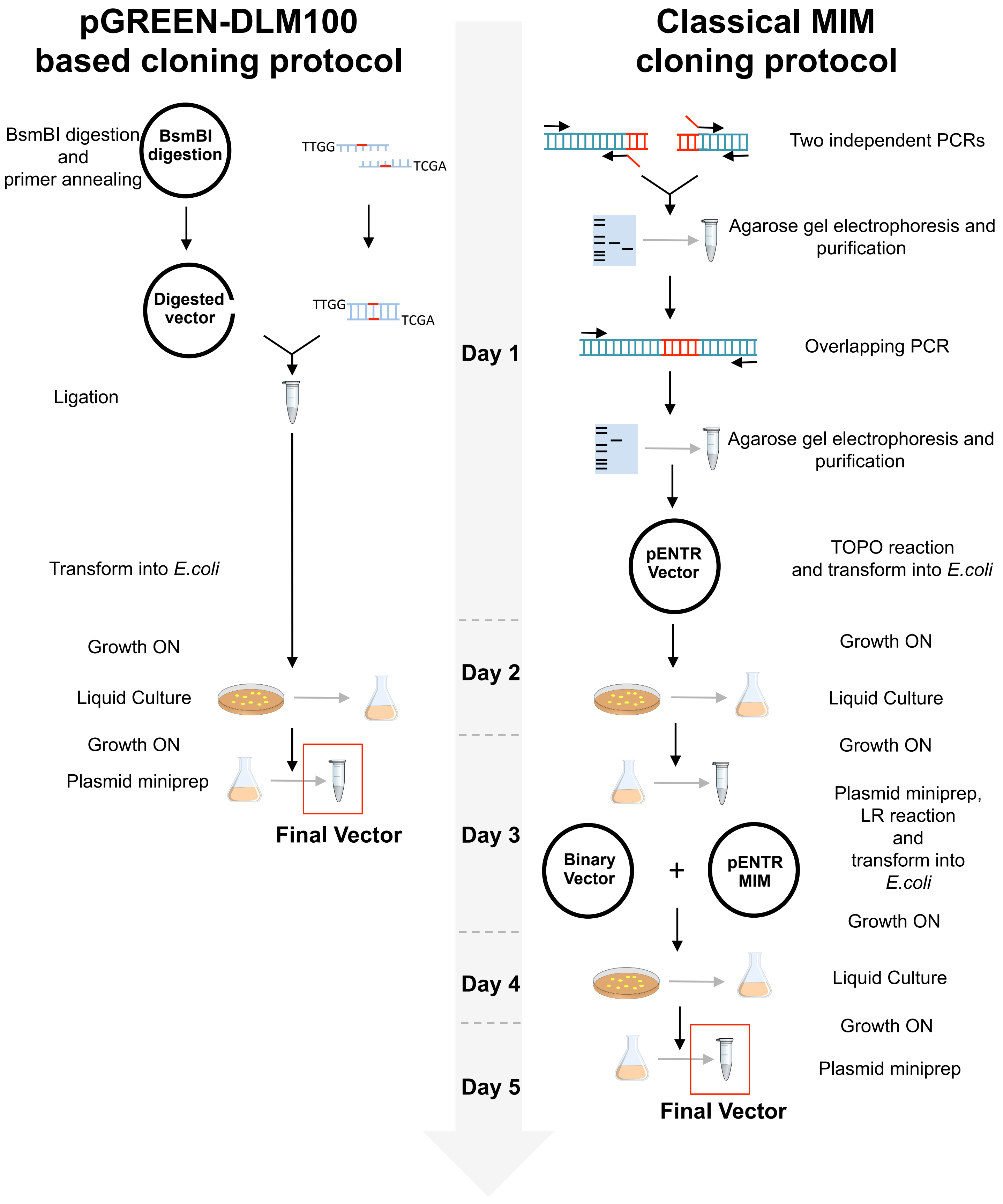

pGREEN-DLM100 based cloning protocol
Classical MIM cloning protocol
BsmBI digestion and primer annealing
BsmBI digestion
TTGG
TCGA
Digested vector
Ligation
Transform into E.coli
Growth ON
Liquid Culture
Growth ON
Plasmid miniprep
Final Vector
Day 1
Day 2
Day 3
Day 4
Day 5
Two independent PCRs
Agarose gel electrophoresis and purification
Overlapping PCR
Agarose gel electrophoresis and purification
pENTR Vector
TOPO reaction and transform into E.coli
Growth ON
Liquid Culture
Growth ON
Plasmid miniprep, LR reaction and transform into E.coli
Binary Vector
+
pENTR MIM
Growth ON
Liquid Culture
Growth ON
Plasmid miniprep
Final Vector
